# Emotions and mathematics: anxiety profiles and their influence on arithmetic performance in university students

**DOI:** 10.1098/rsos.230861

**Published:** 2023-10-11

**Authors:** Serena Rossi, Iro Xenidou-Dervou, Krzysztof Cipora

**Affiliations:** Centre for Mathematical Cognition, Loughborough University, Loughborough, UK

**Keywords:** mathematics anxiety, general trait anxiety, test anxiety, latent profile analysis, self-concept and self-efficacy, arithmetic performance

## Abstract

Mathematics anxiety (MA), general and test anxieties affect mathematics performance. However, little is known about how different anxiety profiles (i.e. individual configurations of anxiety forms) influence the relationship between MA and mathematics performance in university students. To the best of our knowledge, studies that have categorized participants based on their anxiety profiles and investigated how such groups differ in mathematics performance and other individual characteristics have all been conducted only with children and adolescents. Using latent profile analysis, we identified five different anxiety profiles in UK university students (*N* = 328) based on their MA, test anxiety (TA) and trait general anxiety levels (GA). Beyond extreme profiles (high or low levels in all forms of anxiety), we found groups characterized by more specific anxiety forms (MA profile, TA profile and high anxiety with low MA learning profile). These profiles were differentially related to arithmetic performance (but not the performance in a non-mathematics task), and individual factors (e.g. self-concept and self-efficacy). Results can inform the design of interventions tailored to individuals' unique anxiety profiles and highlight the necessity to further study the underpinning mechanisms that drive the MA developmental trajectory from childhood to adulthood.

## 1. Introduction

Mathematical skills are involved in many aspects of our everyday life, such as managing a monthly budget and paying for things in shops. Furthermore, from primary up to high school, mathematics is fundamental to every student's education. However, many people encounter difficulties in mathematics [1]. These difficulties are attributed not only to cognitive (e.g. [2]) but also to emotional factors, such as mathematics anxiety (MA) (e.g. [3,4]).

A considerable proportion of children and adults experience MA at least to some degree, which is related to disruption in their mathematics performance, and avoidance behaviours towards the subject [3]. In fact, mathematics-anxious individuals tend to avoid additional mathematics courses, and they obtain lower grades in those they attend [5,6] regardless of their actual mathematics ability [7]. Therefore, experiencing MA through childhood and adolescence can influence future career choices and success at the university level [5]. In particular, university is a time of high academic pressure. To achieve the degree that is necessary to pursue the career of their choice, students face a series of high-stakes examinations. Although some university students decide not to pursue a mathematics-related degree, mathematics and more specifically arithmetic skills are necessary for many other subjects—e.g. psychology, geography, art, MBA studies, etc.—[8]. Given the negative effects of MA also on university students [9], it is important to further investigate it in this population.

At the same time, accumulating evidence shows that relations between MA and arithmetic performance are not present in a vacuum—other individual characteristics (e.g. other forms of anxiety, personality traits, attitudes) and even more so their constellations seem to have an effect on this link [10,11]. However, so far this has been mostly investigated in children and adolescents, and a comprehensive perspective of relationships between these constructs in university students is still lacking. Moreover, quite often existing studies focus on demonstrating links and relations between constructs that are hypothesized to be correlated while they miss demonstrating the specificity of these relationships. That is, they do not include measures of discriminant validity to demonstrate that certain effects are not present, where they should not be (e.g. that MA is not related to performance in a non-mathematical task of similar characteristics to a mathematical one).

### 1.1. Mathematics anxiety, other forms of anxiety and mathematics performance

MA is defined as ‘a feeling of tension and anxiety that interferes with the manipulation of numbers and the solving of mathematical problems in […] ordinary life and academic situations' [12]. A variety of studies have reported a negative association between MA and mathematics performance with an effect size of around −0.30 (e.g. [13,14]). This negative association appears to become stronger with age, from childhood to adulthood, and it seems to be consistent across all areas of mathematics [13], with a stronger effect of MA on calculations (such as arithmetic) than on geometry. Gender difference is another important aspect to consider in MA research; women generally show a higher level of MA compared to men (e.g. [15–17]).

Beyond MA though, students can be burdened by other forms of anxiety such as test anxiety (TA) and general trait anxiety (GA) [18]. TA is an emotional, physiological and behavioural reaction to potential failure in an academic evaluation [19]. Therefore, TA concerns emotional reactions typically accompanying situations where one's performance is being measured or assessed [20]. On the other hand, GA refers to an individual's predisposition to worry about many different events, behaviours or personal abilities of everyday life [21]. It consists of a feeling of tension, apprehension and increased autonomic reactions, and it is seen as a relatively stable personality trait [22]. Women tend to report higher levels than men not only in MA but also in GA [23] and TA [24,25].

Even though TA and GA also affect mathematics performance, their effect is weaker than the effect of the MA [18,26]. Meta-analyses revealed that GA is more strongly related to TA than to MA, and TA is more related to MA than GA to MA [14,27,28]. Despite the evident relationship between these three constructs, the impact of their concurrent existence on an individual remains unclear.

To examine the effects of different variables on an outcome measure, most studies use multiple regression or latent variable modelling, which considers the co-occurrence of these characteristics at the population level (i.e. variable-centred approach). These methods assume that investigated characteristics are similarly related in every individual [10]. Instead, one may focus on whether groups of individuals revealing specific configurations of characteristics can be identified, and whether these groups differ in terms of the outcome measure. For instance, despite a general negative correlation between two characteristics, there might be some individuals, who score high in both, and their performance in the outcome measure may differ from those who are high in one characteristic and low in the other. To identify such groups from a more heterogeneous population an empirical method called latent profile analysis (LPA) can be used also in the context of numerical cognition and anxiety research [29–32], and it can be seen as an integration of person- and variable-centred analysis.

Carey *et al.* [10] and Mammarella *et al.* [11] used the LPA to examine the impact of concurrent levels of different forms of anxiety in children and adolescents. They found that some individuals may have higher level/s of one or two forms of anxiety compared to others one/s. This means that people could have different configurations/patterns of anxiety that can differently interact with personal and environmental characteristics [10]. Both Carey *et al*.'s [10] and Mammarella *et al*.'s [11] studies identified different groups/profiles of individuals based on their levels of MA, GA and TA. The study by Carey *et al.* [10] involved two age groups of children (9 and 12–13 years old). In the younger children, the authors identified four profiles characterized by low, medium–low, medium–high and high levels of all three forms of anxiety. On the other hand, in the older group (12–13 years old), they identified two profiles characterized by ‘High anxiety’ and ‘Low anxiety’ (respectively the highest and the lowest scores in all three anxiety measures), and two other profiles characterized by the differentiation between the level of different forms of anxiety (specific anxiety forms); a ‘general anxiety’ profile (higher GA but lower MA and TA), and an ‘academic anxiety’ profile (higher MA and TA, but lower GA). Given the well-known gender differences in the considered anxiety forms, the authors also investigated if gender influenced the likelihood of belonging to a particular anxiety profile. In the older group (12–13-year-old adolescents), girls were more likely to be in the ‘general anxiety’ and ‘high anxiety’ profiles, while boys were in the ‘low anxiety’ and ‘academic anxiety’ profiles [10]. Furthermore, they investigated whether profiles differed in mathematics performance and in a reading task. Interestingly, they found that adolescent students (12–13 years old) with high levels of all three anxiety forms (MA, GA and TA) performed better in mathematics than children with specifically elevated (albeit not the highest) academic anxiety (only MA and TA) [10]. This result suggests that the effect of MA on mathematics performance may depend on the concurrent level of other forms of anxiety in an individual. The more dominant one's MA, the larger its potential impact on mathematics performance [10]. However, Mammarella *et al.*'s [11] study did not replicate these results. They considered children in a unique continuous range of ages from 8 to 12 years old, and they identified three profiles: ‘low risk’, ‘average risk’ and ‘high risk’—respectively, low, medium and high scores in all three anxiety measures. Moreover, they did not consider any performance measures. Therefore, even among children and adolescents, the existence of heterogeneous profiles (i.e. ones with different levels of different anxiety types) remains unclear.

To the best of our knowledge, no previous studies investigated the anxiety profiles in university students. In adult university students, we may expect to find more profiles characterized by the differentiation between the levels of the different forms of anxiety, compared to those found in adolescents by Carey *et al.* [10]. MA increases and becomes even more differentiated from general anxiety with age [3]. Moreover, the relationship between MA and mathematics performance seems to be reciprocal, and it has been described as a vicious cycle: poor mathematics performance can trigger MA in some individuals, and this, in turn, can further reduce their mathematics performance [33]. University students will have experienced this vicious circle for longer than younger students, and this could have amplified the developmental trajectory of MA even more. Further, different profiles may have even more differential effects on mathematics performance in adults than in adolescents. Therefore, this warrants the necessity of looking into anxiety profiles in university students.

Mathematical performance/skills in adults have been operationalized differently across studies. Among them, there are one's arithmetic abilities [34]. Arithmetic is widely used in daily life in adulthood [35], and it is strongly affected by MA [17]. Arithmetic is not a unitary ability; it requires many different skills, such as procedural, factual and conceptual skills [36]. There are large individual differences in arithmetic even in adults, including university students. Some struggle with arithmetic, while others have good or rather exceptional calculation abilities [37]. Furthermore, anxiety is linked to lower performance in timed tasks. An arithmetic fluency task, apart from its mathematical component, can be seen as a speeded cognitive task. Therefore, to be able to discriminate the relationship between MA and arithmetic from the relationship between anxiety and speeded cognitive tasks in general, we need to use discriminant validity tasks.

To sum up, not only MA but also other types of anxiety (GA and TA) can affect arithmetic performance. There is some evidence that individual configurations of forms of anxiety may have specific effects on arithmetic performance, beyond the single effect of each anxiety form. However, this requires further research, especially in university students. Anxiety profiles may be also linked to other variables, and this area remains largely unexplored. In the following sections, we review variables which may be relevant both for anxiety profiles and arithmetic performance.

### 1.2. Neuroticism

The anxiety forms so far mentioned are considered personality traits, characterized by a lasting tendency to constantly worry in general (GA, [21]) or about specific types of situations (TA, [38]; MA, [12]). Traditionally, (general) trait anxiety is considered a component of the personality trait of neuroticism [39,40], defined as the tendency to experience frequent, intense negative emotions associated with a sense of uncontrollability—the perception of inadequate coping—in response to stress [41].

There are no previous studies which investigated whether neuroticism is higher in individuals who have high levels of different forms of anxiety concurrently, or whether a high level of neuroticism can also be found in individuals with only one specific/dominant form of anxiety. The address of this issue is of extreme importance since neuroticism has been recognized as a fundamental domain of the personality that has extensive public health implications because it impacts a vast collection of psychopathological and physical healthcare concerns [42]. Therefore, beyond its effect on academic subjects at school, it can also have more damaging life well-being consequences that need to be taken into consideration. As another piece of evidence for the external validity of the profiles, one can thus assume that profiles characterized by high levels of all the trait forms of anxiety considered and dominant GA are characterized by higher neuroticism levels than the profiles with low anxiety and profiles with more specific academic anxiety.

### 1.3. State anxiety

Beyond trait aspects, the anxiety-performance link seems to be also driven by state anxiety—temporary anxiety related to a specific situation [43]. In particular, the relationship between MA and arithmetic performance seems to be more situational [44]. Research suggests that state anxiety can take up available cognitive resources (e.g. working memory) necessary to perform a given task, thus having a direct impact on performance [45,46]. Also, state anxiety can be elicited by a speeded mathematics task because of the pressure created by the time limit for performing the task [47]. However, there is not much research on specific subtypes of state anxiety (state anxiety related to a mathematical task, or state anxiety related to a non-mathematical task). Therefore, it is still unclear whether the level of state anxiety during an arithmetic task is higher in individuals with a dominant trait-MA level profile compared to individuals with all the forms of anxiety. In other words, whether the level of state anxiety associated with a specific task is different based on the trait anxiety forms individuals possess.

### 1.4. Positive attitude towards mathematics

Besides negative factors, others may mitigate the negative relationship between anxiety and performance. Examples of such factors are one's mathematics self-concept (M-self-concept) and mathematics self-efficacy (M-self-efficacy). M-self-concept is defined as one's beliefs about one's competence in mathematics—‘I am good at maths’ [48], while M-self-efficacy is a belief in one's capabilities to execute a mathematics task, for example, maintaining that, ‘I can do this maths problem’ [49,50]. Both these constructs are positively related to mathematics performance [51,52] and negatively related to MA [27,53].

In addition to the forms of self-concept and self-efficacy specifically related to mathematical contexts, there are broader forms of these individual characteristics, such as general self-concept (G-self-concept) and general self-efficacy (G-self-efficacy). G-self-concept is defined as a person's perception in a certain domain, influenced by the evaluation of other people, reinforcement and attributions for one's own behaviour [54]. On the other hand, G-self-efficacy regards one's conviction or belief about their capability to achieve particular results [55]. Most of the previous studies in the context of mathematics did not inspect, using discriminant validity measures (i.e. G-self-concept and G-self-efficacy), whether MA is specifically related to M-self-concept and self-efficacy or also to some general forms of them.

### 1.5. The present study

The present study aimed to identify distinct individual profiles in adult university students based on their level of different forms of trait anxiety (MA, GA and TA), using LPA [10,11].

We investigated whether people in different profiles perform differently in a speeded arithmetic task. To ensure that profiles are not related to performance in every speeded cognitive task, we used a speeded non-mathematics task as a measure of discriminant validity (i.e. a grammatical reasoning task). Furthermore, we examined whether participants classified under different profiles would report different levels of neuroticism, state anxiety assessed immediately after the arithmetic task, and after the non-mathematics task, and positive attitudes towards mathematics (self-concept and self-efficacy). Moreover, we aimed to investigate whether gender influences the likelihood of belonging to one profile rather than another.

We expected to find similar profiles to those found by Carey *et al.* [10] in 12–13-year-old adolescents—i.e. a profile with high levels and one with low levels of all forms of anxiety, and profiles with more specific forms of anxiety, such as one with dominant GA, and one with dominant academic anxiety (MA and TA). However, since this is the first study involving university students, we could not exclude finding different patterns of profiles, such as a profile with a dominant MA, and in general more differentiated profiles than those identified in children and adolescents.

Regarding the comparison of the profiles in different constructs, we expected a lower arithmetic performance in the profile with dominant MA, compared to the profile with a high level of all three forms of anxiety [10]. On the other hand, we did not predict poor performance in the non-mathematics task in this MA profile (e.g. [27,56]). However, we expected poor performance in both arithmetic and non-mathematics grammatical reasoning tasks in a potential academic anxiety profile (high MA and TA).

We predicted the level of neuroticism to be higher in the profile with high levels of all three forms of anxiety, or a potential profile with dominant GA since anxiety seems to be enclosed in the personality trait of neuroticism [39,40]. Then, we expected higher levels of state anxiety (assessed both after the arithmetic and grammatical reasoning tasks) in the profiles with high trait anxiety levels (all forms), in the potential GA profile and in the academic anxiety profile [22] compared to profiles with low trait anxiety levels. However, we predicted higher state anxiety assessed after the arithmetic task in the dominant MA profile compared to the others.

Finally, we expected lower levels of G-self-concept and G-self-efficacy in the profile with high levels of all anxiety forms compared to groups with more specific forms of anxiety. On the other hand, since MA is specifically related to mathematics [57], we predicted finding lower M-self-concept and M-self-efficacy in the group with dominant MA compared to groups with high levels of all anxiety forms.

Regarding gender, we hypothesized that women are more likely to belong to the profile with high levels of all forms of anxiety, and in the profile with dominant GA, while men in the low anxiety group (low anxiety levels in all the forms of anxiety) and academic anxiety group, as found in Carey *et al.* [10] with adolescents. However, since our study involves adult students, we could not exclude different outcomes.

## 2. Method

This study was approved by the Loughborough University Ethics Approvals (Human Participants) Sub-Committee and was then preregistered in the Open Science Framework (OSF) (https://osf.io/nk4ud).

### 2.1. Participants

Participants were students (foundation, undergraduate or master's students) of UK universities recruited via social media (Facebook and Twitter) and Prolific (https://prolific.co/). Only native and fluent English speakers were eligible for the study. A total of 406 participants started the survey. As preregistered, participants were excluded if: (i) they did not complete the entire survey (*N* = 48) [58]; (ii) they were not university students in the UK (*N* = 7); (iii) they stated that they did not speak fluently in English (*N* = 1); (iv) they stated they only wanted to have a look at the survey, without a serious intention to complete it properly (*N* = 5); (v) they responded that their environment was very or extremely noisy during the study completion (*N* = 5); (vi) they declared they did not respond honestly (*N* = 0) and (vii) they spent less than 5 min or more than 40 min on the survey (because of an estimated average completion duration of 15–20 min) (*N* = 9).

The final sample included 331 participants (215 women, 106 men, 7 non-binary gender, 3 no-gender; *M*_age_ = 24.85, s.d. = 7.46) (see sections 4S and 5S in electronic supplementary material in OSF—https://osf.io/62u97/- for the proportions of participants in each macro field of study in the entire sample and divided by men and women). A minimum sample of 300–500 is recommended when using LPA [59–62]. Therefore, our sample seemed sufficient for running the data analyses that we preregistered.

### 2.2. Procedure

The study was conducted online and implemented in Qualtrics Survey (Qualtrics, Provo, UT; see https://osf.io/nk4ud for Qualtrics code of the study). Before starting the survey, participants were asked for informed consent and basic demographic information: age, gender (woman, man, non-binary gender, no-gender, prefer not to say), mother tongue, knowledge of any other languages (if yes, which language/s), and whether they considered themselves fluent English speakers. Details about their educational background (highest educational qualification) and current field of study were also inquired about. Afterwards, participants were asked to perform an arithmetic task and a grammatical reasoning task. After each of these tasks, state anxiety was measured. The order of administration of the arithmetic and the grammatical reasoning tasks with the respective state anxiety questionnaires were counterbalanced between participants. The last part of the survey required the participants to complete the questionnaires on anxiety (MA, TA, GA and neuroticism) and positive individual characteristics (M-self-concept, M-self-efficacy, G-self-concept and G-self-efficacy). Lastly, participants were presented with quality-check items. The total duration of the survey was approximately 20 min.

### 2.3. Materials

In this section, we report the tasks and the questionnaires presented during the study in the administration order.

#### 2.3.1. Performance tasks

##### 2.3.1.1. Arithmetic task

Arithmetic performance was assessed using a speeded calculation task [63,64]. It comprised 40 arithmetic problems presented in one fixed randomized order to be completed with a time limit of 2 min. The 40 problems included all four basic arithmetic operations (addition, subtraction, multiplication and division) and each of them was represented by 10 problems with various difficulty levels (carry/non-carry for additions, borrow/non-borrow for subtractions, part of multiplication table up 10/above 10 for multiplication and divisions). The problems needed to be solved in the given order as it was stated in the instructions. The total score was the sum of the items solved correctly, thus a high score corresponded to high performance.

##### 2.3.1.2. Grammatical reasoning task

Baddeley's grammatical reasoning task [65] tested the understanding of sentences of various levels of syntactic complexity. It consisted of 60 trials. Participants were presented with a series of statements that described the order of presentation of two letters ‘A’ and ‘B’ with the verbs ‘precedes’ and ‘follows’ using two forms of negative and positive and two voices of passive and active e.g. ‘A follows B’—AB (False); ‘B is not preceded by A’—BA (True). Participants had to indicate whether each statement was true or false. The task had a time limit of 3 min and before starting it, participants were presented with four practice trials. The total score was the sum of the items solved correctly, thus a high score corresponded to high performance.

#### 2.3.2. Self-report questionnaires

##### 2.3.2.1. State anxiety

State anxiety was assessed using the Short-STAI questionnaire [66]. This is a five-item questionnaire, which investigates the current state of anxiety. Participants had to state how they feel ‘right now’, by using a 4-point Likert scale. The total score was calculated by summing up responses to all items so that a high score corresponded to high state anxiety. This scale was presented both after the arithmetic and the grammatical reasoning tasks. (Mathematics: Cronbach's *α* = 0.67; Grammatical: Cronbach's *α* = .65 in the current sample).

##### 2.3.2.2. Mathematics anxiety

MA was measured using the abbreviated math anxiety scale (AMAS) [67]. It comprises nine items, divided into two subscales (*Mathematics testing anxiety* and *Mathematics learning anxiety*). Mathematics testing anxiety (items 2, 4, 5 and 8; MA testing) refers to the fear of being evaluated in mathematics, while mathematics learning anxiety (items 1, 3, 6, 7 and 9; MA learning) refers to the fear of learning new mathematical contents. Participants were asked to indicate how anxious certain mathematics situations would make them feel, using a 5-point Likert scale. The total score was calculated by summing up responses to all items so that a high total score corresponded to a high level of MA (Cronbach's *α* was 0.89: Cronbach's *α* Mathematics learning anxiety = 0.83; Cronbach's *α* Mathematics testing anxiety = 0.85 in the current sample).

##### 2.3.2.3. Test anxiety

TA was assessed using the short form of the test anxiety inventory (TAI) [68]. It comprises five items, in which participants are asked to report how frequently they experience specific symptoms of anxiety before, during and after tests and examinations, by using a 4-point Likert scale. The total score was calculated by summing up responses to all items so that a high score corresponded to a high level of TA (Cronbach's *α* = 0.89 in the current sample).

##### 2.3.2.4. General trait anxiety

GA was measured using the GAD-7 [69]. It comprises seven items, which explore how often the person has experienced seven different symptoms of anxiety during the last two weeks, using a 4-point Likert scale. The total score was calculated by summing up responses to all items so that a high score corresponded to a high level of GA (Cronbach's *α* = 0.90 in the current sample).

##### 2.3.2.5. Neuroticism

Neuroticism was assessed using the short version of a neuroticism subscale of the Big Five Inventory [70]. It consists of two items, in which participants respond by using a 5-point Likert scale. They had to indicate the level of agreement for each of the two statements (How well do the following statements describe your personality: (i) ‘is relaxed, handles stress well’; (ii) ‘get nervous easily’). The total score was calculated by summing up responses to the two items (item 1 is reverse-coded) so that a high score corresponded to a high level of neuroticism (Cronbach's *α* = 0.67 in the current sample).

##### 2.3.2.6. Mathematics self-concept

The ‘mathematical ability’ subscale of the self-description questionnaire (SDQ) III [71] was used to investigate participants' M-self-concept. The scale comprises four statements regarding one's ability in mathematics, e.g. ‘I am good in math’. Participants indicate on a 4-point Likert scale to which extent they agree with the statements. The total score was calculated by summing up responses to all items (item 2 and item 4 are reverse-coded), thus a high score corresponded to a high level of M-self-concept (Cronbach's *α* = 0.89 in the current sample).

##### 2.3.2.7. Mathematics self-efficacy

M-self-efficacy was tested using the M-self-efficacy items from the PISA 2003 study (see Lee [72] for the specific items). It consists of six items, in which participants respond using a 4-point Likert scale on how confident they usually feel about mathematics-related situations. The total score was calculated by summing up responses to all items so that a high score corresponded to a high level of M-self-efficacy (Cronbach's *α* = 0.86 in the current sample).

##### 2.3.2.8. General self-concept

G-self-concept was measured using the ‘general self-concept’ subscale of the SDQ III [71]. The scale comprises six statements regarding ability in general. Participants indicate on a 4-point Likert scale to which extent they agree with the statements. The total score was calculated by summing up responses to all items (item 6 is reverse-coded), thus a high score corresponded to a high G-self-concept (Cronbach's *α* = 0.87 in the current sample).

##### 2.3.2.9. General self-efficacy

G-self-efficacy was assessed using the general self-efficacy scale (GSE) [73]. It consists of 10 items, in which participants respond using a 4-point Likert scale on how true each statement was for them. The total score was calculated by summing up responses to all items so that a high score corresponded to a high level of G-self-efficacy (Cronbach's *α* = 0.78 in the current sample).

#### 2.3.3. Quality-check items

Participants responded also to some quality-check items. Before the survey, they had to choose between two alternatives: ‘I want to take this survey seriously’ and ‘I want to have a look at how this survey looks like’. At the end of the survey, participants had to report the level of noise in the environment during the completion of the survey from ‘silent’ to ‘extremely noisy’. Finally, they were asked whether they completed the survey honestly.

### 2.4. Statistical analysis

Statistical analyses conducted for this study have been preregistered in advance and for a detailed description, we refer the reader to the preregistration posted on the OSF (https://osf.io/nk4ud). In summary, we ran Bayesian correlations in the entire sample and also divided by men and women. After that, we conducted LPAs considering the different forms of anxiety as dependent variables or factors in the models (MA testing, MA learning, GA, TA). We deviated from the preregistration at this stage so that we considered the two MA subscales separately in our model instead of considering MA as a unique factor. We made this choice since most of the studies reporting the validation of the questionnaire we are using (AMAS) found that MA is better described by considering the two subscales separately (but related between them) than considering it as a unique factor.

We then compared the obtained groups on different abilities, and emotional and personal characteristics using several one-way ANOVAs. In the subsequent step, we investigated the likelihood of genders belonging to each LPA profile by calculating *χ*^2^ tests and comparing the proportion of women and men between the profiles. Finally, we ran two-factorial between-subjects ANOVAs, with Gender and Profile as independent variables and arithmetic and grammatical reasoning as dependent variables. Data analyses were conducted using R statistical software. For LPA we used the ‘TidyLPA’ package [74]. Data files and R data analysis scripts are available at https://osf.io/62u97/ [75].

## 3. Results

Table 1 reports the descriptive statistics (means and standard deviations) of each considered construct in the entire sample and separately for each gender. Table 2 reports the Bayesian correlations between the considered constructs in the entire sample, while the correlations divided by gender are reported in electronic supplementary material, sections 1S and 2S in OSF—https://osf.io/62u97/.

**Table 1.** Descriptive statistics of all the constructs in the entire sample and divided by genders.

	total *N* = 331 *M* (s.d.)	men *N* = 106 *M* (s.d.)	women *N* = 215 *M* (s.d.)	non-binary *N* = 7 *M* (s.d.)	no-gender *N* = 3 *M* (s.d.)
arithmetic task	13.9 (7.46)	17.80 (9.06)	12.01 (5.68)	14.57 (7.91)	9.67 (5.69)
grammatical task	31.38 (13.37)	31.31 (14.14)	30.98 (13.00)	37.71 (10.70)	47.33 (5.51)
MA	22.98 (7.40)	19.81 (6.59)	24.52 (7.25)	21.00 (8.43)	29.00 (8.54)
MA learning	9.75 (4.10)	8.50 (3.54)	10.36 (4.21)	8.29 (3.73)	13.23 (5.13)
MA testing	13.23 (4.10)	11.32 (3.77)	14.16 (3.85)	12.71 (5.02)	15.77 (3.51)
state anxiety gram	9.37 (2.58)	9.11 (2.61)	9.52 (2.52)	9.43 (2.23)	7.33 (2.08)
state anxiety math	9.40 (2.61)	8.87 (4.47)	9.66 (2.66)	9.29 (2.36)	9.67 (2.08)
general trait anxiety (GA)	15.40 (5.27)	13.65 (4.54)	16.21 (5.43)	17.00 (5.16)	15.67 (4.16)
test anxiety (TA)	14.98 (3.78)	13.48 (3.87)	15.73 (3.57)	14.86 (3.24)	14.67 (2.08)
neuroticism	6.93 (2.04)	6.11 (2.06)	7.28 (1.92)	8.29 (2.06)	8.00 (1.00)
M-self-concept	11.11 (3.01)	12.09 (2.52)	10.61 (3.07)	11.14 (4.88)	12.33 (1.53)
M-self-efficacy	17.76 (4.14)	20.05 (3.36)	16.64 (4.00)	18.00 (5.69)	17.00 (2.65)
G-self-concept	17.74 (3.23)	17.85 (3.07)	17.75 (3.27)	16.29 (4.42)	16.33 (3.21)
G-self-efficacy	28.22 (4.48)	29.42 (4.33)	27.67 (4.43)	28.00 (5.80)	26.00 (3.46)

**Table 2.** Bayesian correlations and credible intervals between all the considered constructs in the entire sample. *p < 0.05.

		1	2	3	4	5	6	7	8	9	10	11	12
1. arithmetic	*r*	—											
	BF10	—											
	CI [95%]	—											
2. grammatical	*r*	0.28*	—										
	BF10	>10 000	—										
	CI [95%]	0.17; 0.37	—										
3. MA	*r*	−0.34*	−0.03	—									
	BF10	>10 000	0.08	—									
	CI [95%]	−0.43; −0.24	−0.14; −0.07	—									
4. state grammatical	*r*	−0.02	−0.11	0.33*	—								
	BF10	0.07	0.49	>10 000	—								
	CI [95%]	−0.12; 0.09	−0.21; −0.01	0.23; 0.42	—								
5. state math	*r*	−0.18*	−0.08	0.42*	0.78*	—							
	BF10	15.95	0.20	>10 000	>10 000	—							
	CI [95%]	−0.28; −0.07	−0.19; 0.02	0.32; 0.51	0.74; 0.82	—							
6. general trait anxiety (GA)	*r*	−0.12	−0.02	0.41*	0.53*	0.54*	—						
	BF10	0.67	0.07	>10 000	>10 000	>10 000	—						
	CI [95%]	−0.22; −0.01	−0.09; 0.12	0.32; 0.50	0.45; 0.61	0.47; 0.62	—						
7. test anxiety (TA)	*r*	−0.26*	−0.09	0.53*	0.27*	0.33*	0.47*	—					
	BF10	4175.75	0.29	>10 000	13094.23	>10 000	>10 000	—					
	CI [95%]	−0.35; −0.15	−0.19; 0.01	0.45; 0.61	0.16; 0.36	0.23; 0.42	0.38; 0.54	—					
8. neuroticism	*r*	−0.15*	0.07	0.41*	0.28*	0.35*	0.53*	0.48*	—				
	BF10	2.71	0.17	>10000	>10 000	>10 000	>10 000	>10 000	—				
	CI [95%]	−0.25; −0.04	−0.03; 0.18	0.31; 0.49	0.18; 0.38	0.25; 0.43	0.44; 0.60	0.39; 0.55	—				
9. M-self-concept	*r*	0.40*	0.21*	−0.61*	−0.16*	−0.26*	−0.16*	−0.31*	−0.20*	—			
	BF10	>10 000	136.18	>10 000	4.31	5649.53	6.09	>10 000	60.39	—			
	CI [95%]	0.30; 0.48	0.11; 0.31	−0.67; −0.54	−0.26; −0.05	−0.36; −0.15	−0.27; −0.06	−0.41; −0.22	−0.30; −0.09	—			
10. M-self-efficacy	*r*	0.43*	0.12	−0.52*	−0.11	−0.19*	−0.17*	−0.35*	−0.30*	0.65*	—		
	BF10	>10 000	0.74	>10 000	0.56	20.91	7.69	>10 000	>10 000	>10 000	—		
	CI [95%]	0.33; 0.51	0.01; 0.22	−0.59; −0.44	−0.21; −0.01	−0.29; −0.08	−0.27; −0.06	−0.44; −0.25	−0.40; −0.20	0.58; 0.70	—		
11. G-self-concept	*r*	0.08	0.02	−0.37*	−0.22*	−0.24*	−0.36*	−0.31*	−0.34*	0.33*	0.21*	—	
	BF10	0.19	0.07	>10 000	250.69	1043.86	>10 000	>10 000	>10 000	>10 000	87.76	—	
	CI [95%]	−0.03; 0.18	−0.09; 0.13	−0.45; −0.27	−0.32; −0.12	−0.33; −0.13	−0.45; −0.26	−0.40; −0.21	−0.43, −0.23	0.22; 0.42	0.10; 0.31	—	
12. G-self-efficacy	*r*	0.13*	−0.09	−0.36*	−0.20*	−0.21*	−0.37*	−0.34*	−0.48*	0.29*	0.30*	0.68*	—
	BF10	1.16	0.23	>10000	39.32	119.70	>10 000	>10 000	>10 000	>10 000	>10 000	>10 000	—
	CI [95%]	0.02; 0.23	−0.19; 0.02	−0.45; −0.27	−0.29; −0.09	−0.31; −0.11	−0.45; −0.27	−0.43; −0.24	−0.56; −0.39	0.19; 0.39	0.19; 0.39	0.61; 0.73	—

### 3.1. Latent profile analysis

Based on the Mahalanobis distance, we excluded three participants classified as extreme outliers for the variables we were considering [76]; therefore, the total final sample included in the LPA was 328. We tested seven models in which we added iteratively one profile each time (from 1 to 7). Table 3 reports fit information for all the tested models.

**Table 3.** Model fit indices for latent profile solutions. *Note:* LL, log likelihood; BIC, Bayesian information criterion; AIC, Akaike information criterion; BLRT *p*-value, *p*-value of the Bootstrapped Likelihood Ratio Test; entropy, value of entropy (from 0 to 1, high entropy corresponds to good model); *N* min, proportion of participants in the smallest profile; *N* max, proportion of participants in the larger profile. The bold highlighted row corresponds to the chosen model.

	LL	BIC	AIC	BLRT p-value	entropy *R*^2^	*N* min	*N* max
*1-Profile*	−1860	3765.63	3735.29	0.01	1.00	1.00	1.00
*2-Profile*	−1697	3469.92	3420.61	0.01	0.76	0.44	0.56
*3-Profile*	−1649	3403.13	3334.86	0.01	0.74	0.25	0.43
*4-Profile*	−1623	3379.45	3292.21	0.01	0.74	0.14	0.37
***5-Profile***	**−1596**	**3353.37**	**3247.16**	**0.01**	**0.79**	**0.09**	**0.33**
*6-Profile*	−1579	3349.99	3224.82	0.01	0.80	0.04	0.27
*7-Profile*	−1570	3360.93	3216.80	0.04	0.81	0.03	0.26

Although the BLRT *p*-values showed in each step a significant difference between profiles from 1 to 7 profiles, the BIC value started to increase in the model with seven profiles, which suggests accepting the model with six profiles. However, the six-profile model showed that the smallest group among the six profiles included only 4% of the entire sample (*N* min = 0.04), which, according to our preregistered criteria, suggests model rejection. Therefore, we accepted the model with five following profiles:

Profile 1: *High anxiety* (high level in all four anxiety forms).
Profile 2: *High anxiety with low MA learning* (high level of all anxiety forms except for the learning subscale of MA which demonstrated a fairly low score).
Profile 3: *MA* (medium levels of GA and TA, but medium/high level of MA—both MA Testing and MA learning).
Profile 4: *TA* (medium level of GA and MA, but medium/high level of TA).
Profile 5: *Low anxiety* (low level in all four anxiety forms).

**Figure 1.**
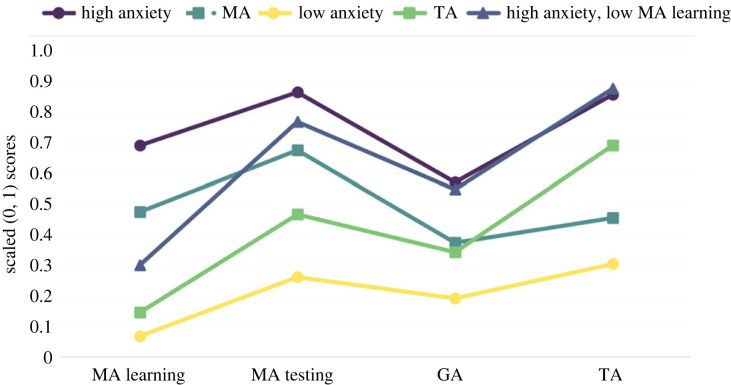
Scaled (0,1) values of each profile in each variable considered in the LPA.

Figure 1 shows the scaled (0,1) values for each profile in each variable considered in the study.

### 3.2. Profile comparisons

**Figure 2.**
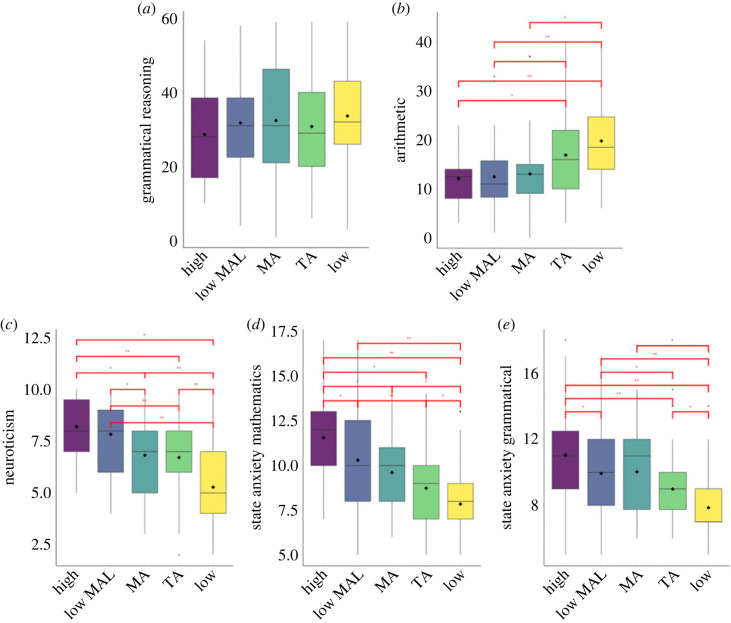
Graphical representation of the comparisons between profiles in performance in (*a*) grammatical reasoning, (*b*) arithmetic, (*c*) neuroticism as well as state anxiety related to (*d*) arithmetic task and (*e*) grammatical reasoning. Note: high = ‘high anxiety’, lowMAL = ‘high anxiety with low MA learning’, MA = ‘MA’, TA = ‘TA’, low = ‘low anxiety’.

Having identified our five profiles, we compared them with respect to the arithmetic and the grammatical reasoning tasks, as well as in their levels of neuroticism, state anxiety immediately after the arithmetic task, state anxiety immediately after the grammatical reasoning task, M-self-concept, G-self-concept, M-self-efficacy, G-self-efficacy. As reported in table 4, one-way ANOVAs revealed that the only non-significant comparison was the one in the grammatical reasoning task (*p* = 0.370), showing that profile membership was not related to the performance in this task (figure 2*a*). *Post hoc* comparisons showed that the students in the ‘high anxiety’, the ‘high anxiety with low MA learning’ and the ‘MA’ profiles demonstrated the lowest (and similar between them) arithmetic performance. On the contrary, individuals in the ‘low anxiety’ profile exhibited the highest arithmetic performance. Students in the ‘TA’ profile performed worse than the ‘low anxiety’ profile, and better than the ‘MA’ profile’, although this last comparison was not statistically significant (figure 2*b*). It is worth noting that only for the arithmetic task the comparison between profiles has been conducted by considering only participants who performed the task in the given order as instructed (see Method). As an exploratory analysis, we also tested, whether skipping items (therefore, likely trying to perform only the less difficult problems) varied between the anxiety profiles. The *χ*^2^ difference test showed that there was no difference between anxiety profile in the proportion of participants who skipped items during the arithmetic task, $\chi_{4}^{2}=2.68$, *p* = 0.61.

**Table 4.** Descriptive statistics and analysis of variance between profiles. *Note: ‘W’ in the second row of the table refers to the number of women in each profile.

variables	*M* (s.d.)								
	Profile 1 high anxiety *n* = 47(38 W) (14.3%)	Profile 2 highlowMAL *n* = 79(65 W) (24.1%)	Profile 3 MA *n* = 28(20 W) (8.6%)	Profile 4 TA *n* = 109(59 W) (33.2%)	Profile 5 low anxiety *n* = 65(31 W) (19.8%)				
MA learning	16.58 (2.22)	10.15 (2.34)	13.07 (2.07)	7.51 (1.68)	6.18 (1.53)				
MA testing	17.84 (2.05)	16.32 (1.84)	14.82 (2.34)	11.46 (2.30)	8.23 (2.49)				
GA	19.04 (4.49)	18.54 (4.67)	14.86 (5.10)	14.24 (4.19)	11.09 (3.85)				
TA	17.89 (1.71)	18.18 (1.71)	11.82 (1.74)	15.37 (2.07)	9.57 (2.39)				
						ANOVA			
	*M* (s.d.)					*F*-value	*p*-value	$\eta_{p}^{2}$	BF_10_
arithmetic	12.14 (5.69)	12.46 (6.76)	13.05 (7.73)	16.92 (8.22)	19.80 (8.97)	(4,211) = 8.07	< 0.001	0.13	4223.65
grammatical	28.62 (12.61)	31.77 (13.03)	32.39 (14.19)	30.75 (13.43)	33.62 (13.87)	(4, 323) = 1.07	0.370	0.01	0.046
neuroticism	8.21 (1.47)	7.84 (1.70)	6.82 (2.16)	6.72 (1.71)	5.28 (2.02)	(4, 323) = 25.58	< 0.001	0.24	3.22 e^15^
state anxiety math	11.55 (2.38)	10.30 (2.91)	9.61 (2.20)	8.73 (2.08)	7.85 (1.91)	(4, 323) = 22.60	< 0.001	.22	3.78 e^13^
state anxiety gram	11.05 (2.81)	9.94 (2.75)	10.04 (2.71)	8.99 (2.08)	7.86 (1.87)	(4, 322) = 14.64	< 0.001	0.15	1.774 e^18^
M-self-concept	8.19 (2.74)	10.37 (2.54)	9.32 (3.08)	12.17 (2.22)	13.18 (2.39)	(4, 323) = 37.69	< 0.001	0.32	4.96 e^22^
G-self-concept	15.55 (4.24)	16.75 (3.22)	17.54 (2.49)	18.52 (2.54)	19.20 (2.48)	(4, 323) = 14.18	< 0.001	0.15	> 10 000
M-self-efficacy	14.66 (4.01)	16.75 (3.74)	16.21 (3.63)	18.61 (3.44)	20.75 (4.57)	(4, 323) = 24.72	< 0.001	0.23	8.1 e^14^
G-self-efficacy	25.79 (5.10)	26.56 (4.44)	28.39 (4.33)	28.86 (3.49)	30.72 (4.15)	(4, 323) = 13.55	< 0.001	0.14	> 10 000

The highest neuroticism levels were found in the ‘high anxiety’ and ‘high anxiety with low MA learning’ profiles, while the lowest level was in the ‘low anxiety’ profile. Medium levels were observed for the two academic anxiety profiles (‘MA’ and ‘TA’ profiles; figure 2*c*). State anxiety assessed immediately after the arithmetic task and immediately after the grammatical reasoning task showed similar patterns; the highest levels were observed for participants in the ‘high anxiety’ profile, whilst the lowest was in the ‘low anxiety’ profile (figure 2*d,e*).

**Figure 3.**
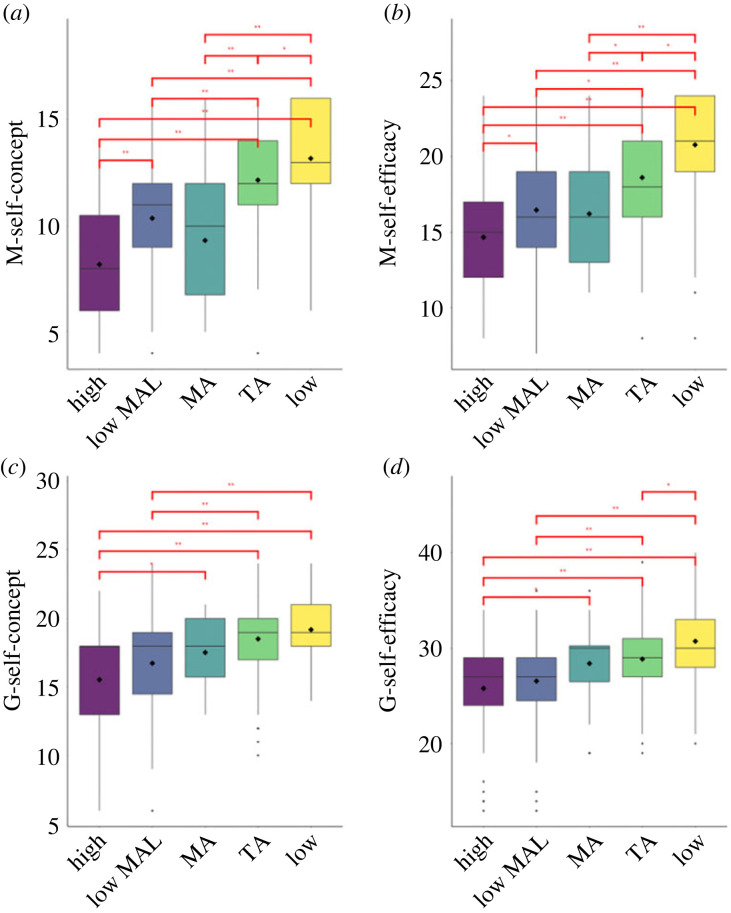
Graphical representation of the comparisons between profiles in self-concept and self-efficacy. (*a*) Mathematics self-concept, (*b*): mathematics self-efficacy, (*c*) general self-concept, (*d*) general self-efficacy. Note: high = ‘high anxiety’, lowMAL = ‘high anxiety with low MA learning’, MA = ‘MA’, TA = ‘TA’, low = ‘low anxiety’.

Regarding positive attitudes, such as M-self- concept, the lowest levels were observed for individuals in the ‘high anxiety’ and ‘MA’ profiles, and these were similar between them. The ‘high anxiety with low MA learning’ individuals reported a significantly higher M-self-concept level than the ‘high anxiety’ individuals, but not than the ‘MA’ ones. Students in the ‘TA’ profile showed a significantly higher M-self-concept level than the ones in the high anxiety and ‘MA’ profiles, but significantly lower than the ‘low anxiety’ profile (figure 3*a*). M-self-efficacy comparison between profiles showed a similar pattern to the M-self-concept (figure 3*b*).

Concerning discriminant validity measures, the two high anxiety profiles students showed the lowest G-self-concept. We then observed a higher level in the two academic anxiety profiles, and a further higher level in the ‘low anxiety’ profile but not significantly higher than the ‘MA’ and ‘TA’ profiles (figure 3*c*). A similar pattern was found for G-self-efficacy but with a significantly higher level in the ‘low anxiety’ profile than in the ‘TA’ profile (figure 3*d*). In table 4, we also reported Bayes factors associated with all ANOVAs, which quantify evidence for null and alternative hypotheses.

### 3.3. Latent profiles and gender

**Figure 4.**
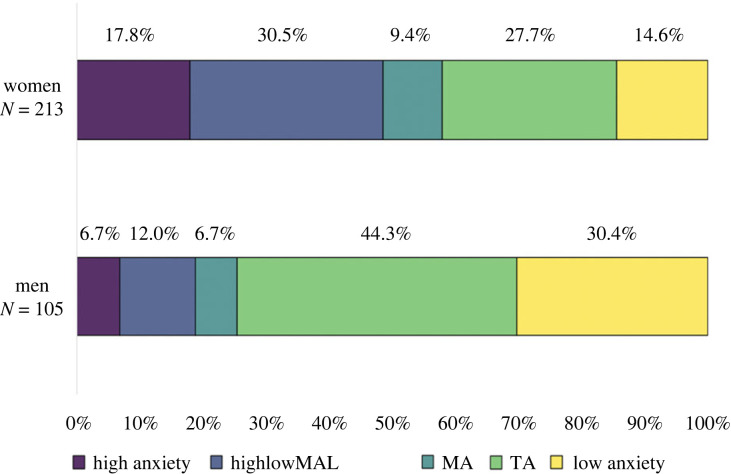
Percentage of men and women in each profile: ‘high anxiety’, highlowMAL = ‘high anxiety with low MA learning’, MA = ‘mathematics anxiety’, TA = ‘test anxiety’, low = ‘low anxiety’.

We then investigated the difference in proportions of individuals belonging to a specific profile based on gender; the *χ*^2^ test of independence was calculated by comparing the frequency of profile membership in men and women. There was a significant difference, $\chi_{4}^{2}=32.54$, *p* < 0.001: Women were more likely to be in the ‘high anxiety’, the ‘high anxiety with low level of MA learning’ and the ‘MA’ profiles, while men were more likely to belong in the ‘low anxiety’ and the ‘TA’ profiles (cf. Figure 4).

Finally, the two-factorial between-subjects ANOVA with Profile and Gender (only men and women) as independent variables and arithmetic performance as a dependent variable showed a significant main effect of Profile, *F*_4,198_ = 2.67; *p* = 0.03; $\eta_{p}^{2}=0.05$, and a significant main effect of Gender, *F*_1,198_ = 26.53; *p* < 0.001; $\eta_{p}^{2}=0.12$, while the interaction between the two factors was not significant, *F*_4,198_ = 1.67; *p* = 0.16; $\eta_{p}^{2}=0.03$. We observed higher arithmetic performance in men compared to women within all five profiles. Regarding the grammatical reasoning task, there were no main significant effects of Profile, *F*_4,308_ = 1.28; *p* = 0.28; $\eta_{p}^{2}=0.02$, or Gender, *F*_1,308_ = 0.39; *p* = 0.53; $\eta_{p}^{2}=0.001$, nor significant interaction, *F*_4,308_ = 1.5; *p* = 0.20; $\eta_{p}^{2}=0.02$. Descriptive statistics for each profile in each variable for men and women separately are reported in electronic supplementary material, section 3S in OSF—https://osf.io/62u97/).

## 4. Discussion

It is well known that there is a negative association between MA and mathematics performance [13,14]. However, other forms of anxiety also affect mathematics performance [26,77–79]. This study investigated whether we could identify distinct individual profiles in adult university students based on their level of different forms of anxiety (MA, GA, TA) and if these different anxiety profile groups performed differently in an arithmetic task and a non-mathematics speeded task (grammatical reasoning task). Further, it investigated whether profiles differed in terms of the levels of neuroticism, state anxiety assessed immediately after the arithmetic and the non-mathematics tasks, and levels of several positive personality aspects/beliefs. Moreover, we examined the proportions of individuals belonging to a specific profile based on gender and the potential differences in arithmetic and grammatical reasoning performances between genders in each profile. To the best of our knowledge, this is the first study that identified and examined these anxiety profiles in university students—a stage in life where academic performance crucially influences one's future career. We found five different anxiety profiles. These profiles were differently related to arithmetic performance (but not the performance in the non-mathematics grammatical task) and individual factors, such as self-concept and self-efficacy.

### 4.1. Latent profile analysis

To identify different profiles, we conducted LPA considering the students' levels of MA learning, MA testing, TA and GA. Results revealed five distinct profiles in our sample. We observed: (i) individuals with high levels of all forms of anxiety (*high anxiety* profile), (ii) individuals with low levels of all forms of anxiety (*low anxiety* profile), (iii) a group with high levels of GA, TA and MA testing, but a fairly low level of MA learning (*high anxiety with low MA learning* profile). We then found two intermediate profiles: (iv) medium/high levels of MA (both MA testing and MA learning) but lower levels of GA and TA (*MA* profile) and (v) medium/high level of TA but lower levels of GA and MA (*TA* profile).

These findings are partly in line with our hypotheses based on Carey *et al.*’s [10] results. They found different profile patterns between a group of 9-year-old and a group of 12–13-year-old children in the UK: besides the extreme groups, they also found a profile with differentiation in the level of anxiety forms. Specifically, a profile with higher GA and lower MA and TA (‘general anxiety’ profile), and a profile named ‘academic anxiety’ with higher TA and MA and lower GA. Our results showed an even greater differentiation between the academic anxiety forms—a profile with a specific higher MA, and a profile with a specific higher TA, compared to adolescents. A reason for the observed extended differentiation in anxiety profiles amongst university students could be due to their long experience in and exposure to academic environments, which means potential prolonged exposure to the vicious circle of the relationship between MA and mathematics performance [33]. This could have facilitated the development of a specific/dominant MA for some of these students. On the contrary, individuals with a TA-specific profile may not have experienced this particular vicious circle in the context of mathematics, but instead, may have developed anxiety about test situations in general.

### 4.2. Profiles’ comparisons of performance

In the arithmetic task, students in the ‘high anxiety’ and the ‘high anxiety with low MA learning’ profiles demonstrated the lowest performance, while the students in the ‘low anxiety’ showed the highest one. Unlike Carey *et al.* [10], we did not find a better performance in the high anxiety profiles compared to the ‘MA’ profile in the arithmetic task. Instead, performance was equally low within both of these profiles. This result not only confirms the detrimental effect that MA can have on arithmetic performance but also demonstrates that MA could be the primary form of anxiety that drives the drop in arithmetic performance in the high anxiety profiles too. This conclusion can also be confirmed by the arithmetic performance in the ‘TA’ profile. Although those university students did not perform significantly better than the students in the ‘MA’ profile, their mean raw score (accuracy) was higher than the raw score in the ‘MA’ profile, and it was not significantly lower than the ‘low anxiety’ accuracy. This leads us to conclude that TA could impair mathematics performance, but its role is not as pervasive as the MA. It is noteworthy that the ‘TA’ group was by far the most heterogeneous when it comes to arithmetic performance (figure 2*b*). This on its own would require some future investigation. At the same time, this variability may also account for the lack of significant difference between the ‘TA’ and the ‘MA’ or ‘low anxiety' profiles' performance. Furthermore, as already reported, the ‘high anxiety with low MA learning’ profile, although characterized by high levels of anxiety similar to those in the ‘high anxiety’ profile, exhibited a relatively low MA learning.

Students in this profile also showed poor arithmetic performance as in the ‘high anxiety’ and ‘MA’ profiles. This may further suggest that the MA testing could be driving the effect on arithmetic performance, especially in a timed task as we used in this study [80]. The greater relevance of the MA testing component compared to the MA learning one in influencing arithmetic performance might be intuitive if we consider that we were evaluating—and therefore testing—the students' performance and not their efficiency in learning new mathematical contents, which might be more affected by the MA learning component. In addition, previous studies reported that the testing component of MA plays a primary role in the definition of the overall MA level [81,82]. However, it is worth noting that the difference in our results compared to the ones found by Carey *et al.* [10] in relation to mathematics performance, may also be due to the fact that the arithmetic task we used was still relatively low-stakes for the university students. On the contrary, Carey *et al.* [10] used a 45 min curriculum-based standardized test, which assessed recently taught material. This could have heightened the high-stakes nature of the situation. Therefore, future studies should identify the relevance of the identified profiles within more high-stakes testing for university students, and in the context of no-response errors or task disengagement.

Contrary to our expectations, we did not find differences between the five anxiety profiles in the performance of the grammatical reasoning task. In fact, we expected that the grammatical reasoning task, as a discriminant validity task, would not be specifically affected by the MA profile but by profiles with at least high TA. Importantly, the grammatical reasoning task was similar to the arithmetic task in terms of different levels of difficulty and time limits to perform it. The grammatical reasoning task consisted of sentences with various levels of difficulty (two forms of negative and positive and two voices of active and passive). Similarly, the arithmetic task included all four types of operations (additions, subtractions, multiplication and divisions), each of them with two levels of complexity (carry/non-carry, borrow/non-borrow, multiplication table up to 10/above 10). In both tasks, participants had a time limit to undertake them (2 min to perform 40 trials in the arithmetic task, and 3 min to perform 60 trials for the grammatical one). Also, as observed in figure 2, we did not find a ceiling effect nor a floor effect for any profile in the discriminant validity task. Therefore, we can conclude that the similar performance in the grammatical task across profiles is because the different anxiety patterns did not differentially influence the outcome in this task, and not because of its structural difference with the arithmetic task. At the same time, the grammatical task performance did not correlate with any of the anxiety forms at the group level. However, it correlated with the arithmetic task, which reflects the typically observed positive correlation between different cognitive tasks.

### 4.3. Latent profiles and self-concept and self-efficacy

The ‘high anxiety’ and the ‘MA’ students reported the lowest levels of M-self-concept. The ‘high anxiety with low MA learning’ students showed a significantly higher M-self-concept level than the one in the ‘high anxiety’ profile, but not significantly higher than the level in the ‘MA’ profile. The ‘TA’ individuals reported a significantly higher M-self-concept than students in all the aforementioned profiles, but significantly lower than the ‘low anxiety’ individuals, who demonstrated the highest level.

This is not entirely in line with our expectations that individuals with dominant MA (‘MA’ profile) should have reported lower M-self-concept than individuals with high levels of all the forms of anxiety. Given our results, we can assume that it is the level of MA in individuals that influence their M-self-concept level, rather than its dominance over the other forms of anxiety. Moreover, having lower levels of one component of MA, as in our sample for the ‘high anxiety with low MA learning’, may help avoid an extremely low M-self-concept. This can be further corroborated also by the ‘TA’ profile responses; having a specific TA but a fairly low MA may have prevented a very low M-self-concept in those students. Furthermore, low levels of all forms of anxiety (‘low anxiety’ profile) may be the best configuration for preventing a low M-self-concept. These results confirm the idea that MA could be especially relevant in shaping one's M-self-concept and also in individuals with high levels of all forms of anxiety and not only MA. This assumption is in line with previous studies which found that MA directly influences the M-self-concept in children [83] and university students [64].

Regarding M-self-efficacy we found a similar pattern of differences between the five profiles as the ones found for M-self-concept, confirming the idea that although these are two distinct constructs, they are strongly related (*r* = 0.65). Self-concept is more general and includes judgments of self-worth, while self-efficacy is related to a specific context and concerns the perception of the ability in performing that specific task [52]. Self-concept seems to concern a more stable interpretation of past experiences, while self-efficacy seems to be about a contextual-specific evaluation that focuses on future potentials. Since both constructs focus on self-perception of capabilities, when they are confined to specific domains, these perceptions begin to overlap [54].

Regarding less specific forms of self-concept and self-efficacy, for G-self-concept, we found the lowest levels in the high anxiety profiles (both ‘high anxiety’ and ‘high anxiety with low MA learning’). Higher levels for the two academic anxiety profiles (and similar among them), and a further higher (even if not significant) level for the ‘low anxiety’ profile. A similar pattern of differences has been found for G-self-efficacy with the exception that, in this case, the difference between the ‘TA’ profile and the ‘low anxiety’ profile was also significant (figure 3). These results are in line with our expectations of lower G-self-concept and G-self-efficacy levels in the high anxiety profiles compared to groups with more specific forms of anxiety. Given these outcomes, we can assume that GA, rather than MA, shapes the level of these domain-general variables. In fact, with the decrease of the GA across profiles, G-self-concept and G-self-efficacy increase. This assumption is also supported by the correlations between GA and the G-self-concept and G-self-efficacy values (around −0.30). We can, therefore, conclude that we achieved discriminant validity between mathematics-related and general-related aspects.

### 4.4. Latent profiles, neuroticism and state anxiety

Another more general personality aspect we considered is neuroticism. As expected, the comparison between profiles demonstrated the highest level in the high anxiety profiles, the lowest level in the ‘low anxiety’ profile and intermediate levels in the academic anxiety profiles. This means that the more forms and the higher the levels of anxiety co-existing in an individual, the higher the level of neuroticism reported. This is in line with the existing literature reporting that trait anxiety (in our case also TA and MA are trait forms of anxiety) are considered components of the personality trait of neuroticism [39,40]. At the same time, academic anxiety, which is bound to specific domains may be at least to some degree shaped by individual negative experiences, and not necessarily be driven by general personality characteristics.

To evaluate the anxiety perceived during the survey in relation to our two tasks, we compared the state anxiety assessed immediately after the arithmetic task and immediately after the grammatical reasoning task across the five profiles. In both cases, state anxiety decreased with the decreasing number of trait anxiety forms and their levels among the profile groups, without showing specificity for one task over the other. We can conclude that students in the different profiles did not show a difference in the state anxiety across the tasks. Nevertheless, the questionnaire we presented after both tasks was the same, and the questions presented to the students were quite general. They were asked to state their feelings at that specific moment without specific reference to the task they had just performed. It is possible that using more specific task-related questions could have provided more differentiated responses by the students in the different profiles and different tasks. However, this assumption needs to be further investigated.

### 4.5. The role of gender

Finally, we studied the proportion of individuals in each profile based on gender. Our results showed that women were more likely to belong to the high anxiety profiles (‘high anxiety’ and ‘high anxiety with low MA learning’) and the ‘MA’ profile, while men to the ‘low anxiety’ and ‘TA’ profiles. These results are in line with the existing literature regarding gender differences in MA and GA. Women generally report higher GA [23] and MA than men [15–17], due to a variety of reasons. These include societal beliefs—e.g. mathematics gender stereotypes—[84,85] the possible endorsement of these beliefs [64], lower self-perception and confidence [86–88] and the fact that girls/women are more likely to openly state their negative feelings [6].

Although recent studies reported no difference in mathematics performance between genders (e.g. [15]), we found lower scores in the arithmetic task in women compared to men within all the profiles. If we consider the field of study that our participants declared to be attending at the university (see electronic supplementary material, sections 4S and 5S in OSF—https://osf.io/62u97/), we notice that there is a homogeneous distribution of students studying for university degrees included in the areas of literature, psychology, social sciences and STEM (around 23% of participants in each area). However, considering men and women separately we observed a higher percentage of women in the literature and psychology areas compared to the others. In contrast, we observed a higher percentage of men in STEM and social sciences (which includes also economics where mathematics is crucial) compared to other areas. This reflects the well-known under-representation of women in the mathematics-intensive STEM fields [89]. Thus, the observed poorer arithmetic performance in women compared to men potentially relates to their previous avoidance of or disengagement in mathematics due to societal influences and contextual factors, which led them to choose a non-mathematics-related field of study and career [90]. Moreover, due to their study programme, they may be having less day-by-day practice with mathematics.

It is worth noting that as an exploratory analysis, we tested whether there was a significant difference between STEM and non-STEM programme choices across anxiety profiles. The *χ*^2^ difference indicated no significant difference between anxiety profiles in university programme selection, $\chi_{4}^{2}=4.83$, *p* = 0.31. This means that the type of anxiety profile of the students was not associated with the university degree programme they were attending.

### 4.6. Limitations and strengths of the study

This study has some limitations, such as the fact that the survey was conducted online, which does not give us the same level of control as in a laboratory study. At the same time, the observed pattern of correlations and task reliabilities is in line with what has been reported about these measures/constructs in numerous previous studies across both online and in-laboratory setups.

Moreover, the data collected from the state anxiety questionnaires, which were administered just after both the arithmetic and grammatical reasoning tasks, convey that when filling them, participants were most probably not referring to their anxiety during the given task, but more in general to their state anxiety at that particular moment. This was probably due to the lack of reference to the specific task just performed in the questions posed, therefore future studies should assess state anxiety by retrospectively asking participants how they felt during the preceding (arithmetic or grammatical) task or administering the scale halfway through the respective task.

Additionally, we assessed neuroticism using only the short version of the neuroticism subscale of the Big Five Inventory (two items) [70], and this does not allow us to make strong inferences regarding the results related to this construct. Neuroticism did not play a crucial role in the present study, however, future studies focusing on elucidating the role of this particular construct should aim at using the entire subscale of the questionnaire to obtain more reliable results.

A strength of this study though is the systematic use of discriminant validity measures for both performance and individual factors. This allowed us to have a broader overview of what happens in university students regarding their anxiety and correlated factors, and which relationships are specific to mathematical and non-mathematical content. This is also the first study that has investigated all these aspects together in university students, who are at a crucial stage of their academic and professional development.

## 5. Conclusion

Our study demonstrated how university students in the UK with different anxiety profiles can demonstrate different arithmetic performance and other mathematics- and non-mathematics-related aspects. Importantly, these measures were not considered when the profiles were identified, so the differences we observe cannot be attributed to method artefacts and/or circular reasoning.

Although our results are slightly different compared to the ones found by Carey *et al.* [10] in UK adolescents, we can assume that the explanation they proposed regarding the development of MA is valid also for our results. Some individuals could have developed MA as a general predisposition to anxiety. In our study, these individuals would be the ones in the ‘high anxiety’ and ‘high anxiety with low MA learning’ profiles, who showed poor arithmetic performance along with generally lower levels of self-concept and self-efficacy compared to other profiles. These individuals' general predisposition for anxiety is also evident in their higher levels of neuroticism [41]. On the other hand, other individuals could be less vulnerable to general anxiety but they may possess specific risk factors for certain academic anxiety forms [10], in our case people in the ‘MA’ and ‘TA’ profiles. This idea is also supported by their levels of neuroticism that, even if not low, were nevertheless lower than the ones reported by the highly anxious students. Specifically, individuals in the ‘MA’ profile may have developed only MA due to past repeated poor mathematics performances, with a consequent decrease in mathematics self-perceptions establishing a vicious circle between these elements. Concerning the ‘TA’ profile, these individuals could have a more general predisposition of fear of tests at school but with less relevant consequences in academic self-perception and academic performance. However, all these assumptions need to be further investigated with longitudinal designs.

Given the different impacts of each anxiety pattern on performance and on the individual factors (mathematics-related and non-mathematics-related), this study could help develop different anxiety interventions that take into account individual anxiety profiles. In fact, even if we found similar poor arithmetic performance across some of our anxiety profiles (concurrent forms of anxiety and only dominant MA), our data show that different anxiety patterns also were differentially associated with other individual factors. These factors, if mitigated, could enhance the academic and general well-being of university students [91], who are still shaping their future careers and need to make some important choices for their own future lives. In addition, the differentiation between MA learning and MA testing found in one of our profiles (high anxiety with low MA learning) further confirms the multidimensional nature of MA (e.g. [67]), which should be considered in future studies. This can be particularly important for the development of interventions, which may need to target anxiety in general for individuals with high anxiety profiles or to be more targeted to MA or even its specific components.

The different anxiety patterns/configurations we found in UK university students, compared to those found in UK adolescents by Carey *et al.* [10], indirectly support our initial hypothesis that there could be a developmental change in the differentiation of anxiety profiles. Nevertheless, we need to be cautious in asserting a developmental change because we did not conduct a longitudinal study or include different age groups as in Carey *et al.* [10]. Moreover, the outcome measures (mathematics tasks) in the two studies were different and had different importance for the participants. As already mentioned, the arithmetic task we used in this study could be considered a low-stakes task for university students, who we expect to be able to perform more complicated mathematical tasks. In this regard, the difference in our results compared to the one found by Carey *et al.* [10] may also reflect the self-selection of students who decide to go to study at the university. All of these arguments raise the need to further investigate the underlying causal mechanisms that may drive this differentiation (the development of some specific/dominant forms of anxiety) in some individuals from childhood to adulthood, in order to find a way to avoid it while still young. This would help vulnerable individuals not only increase their chances of a successful future career but also improve their everyday life well-being.

## Data Availability

The Qualtrics code of the study can be found in the Open Science Framework at https://osf.io/nk4ud, as well as the preregistration of the study. The link can be found in the Procedure section in the manuscript. The data files can be found at: https://osf.io/62u97/, as well as the R script of the data analyses. This link can be found in the ‘Statistical analyses’ section in the manuscript.
